# PD14 - Non-interventional 2-year study of sublingual immunotherapy in children and adolescents with allergic rhinoconjunctivitis caused by grass pollen

**DOI:** 10.1186/2045-7022-4-S1-P14

**Published:** 2014-02-28

**Authors:** Efstrathios Karagiannis, Kija Shah-Hosseini, Meike Hadler, Ralph Mösges

**Affiliations:** 1 Stallergenes GmbH, Kamp-Lintfort, Germany; 2 Institute of Medical Statistics, Informatics and Epidemiology (IMSIE), University Hospital Cologne, Cologne, Germany

## Background

The aim of this non-interventional study was to document the impact of a sublingual allergen immunotherapy (AIT) with Oralair 5-grass pollen tablets (Stallergenes, France) on symptom severity, use of symptomatic medication and tolerability in patients with grass pollen-induced allergic rhinoconjunctivitis (RC) over 2 years of routine medical practice treatment. This poster focuses on the subgroups of children (4-11 yrs) and adolescents (12-17 yrs).

## Methods

This prospective, open, non-controlled, non-interventional, multicenter study was conducted from September 2010 to October 2012 in Germany. Overall 1.482 patients (248 children (16.9%), 201 adolescents (13.7%)) participated in the study.

The patients rated their symptoms as a combined score of severity (scale: 0 [none] – 3 [severe]) and frequency (scale: 0 [none] – 4 [very often]). In the combined RC score, the severity of rhinitis and conjunctivitis were pooled (scale: 0 [none] – 6 [severe]).

## Results

During the season preceding AIT treatment 88/ 85% of children/ adolescents had used symptomatic medication. This rate dropped to 57/ 56% (1^st^ season) and to 48/ 40% (2^nd^ season). Likewise the RC score decreased from 4.10/ 4.05 to 1.93/ 1.97 (1^st^ year) and to 1.39/ 1.39 (2^nd^ year).

An improvement in health status after two years of treatment was documented by 96/ 97%.

Adverse events occurred in 17.7/ 20.2% over two years of treatment. The incidence of non-fatal serious adverse events was 1.2/ 0.0%.

At the end of the 2^nd^ season, 96/ 97% evaluated the tolerability of the 5-grass pollen tablets as very good or good.

For the group of monoallergic children/ adolescents, the RC score was reduced from 4.05/ 3.63 to 1.95/ 1.85 (1^st^ year) to 1.29/ 1.10 (2^nd^ year). The RC score in polyallergic children/ adolescents decreased from 4.09/ 4.17 to 1.94/ 2.03 (1^st^ year) to 1.46/ 1.50 (2^nd^ year).

## Conclusion

Based on the study results, AIT with Oralair 5-grass pollen tablets was well tolerated by children and adolescents in routine medical practice. All symptom scores and the symptomatic medication use were reduced significantly after one and two years under treatment with Oralair 5-grass pollen tablets compared to the season preceding AIT. Polyallergic children and adolescents benefited as much from AIT as the monoallergic ones.

